# Modulating the water channel AQP4 alters miRNA expression, astrocyte connectivity and water diffusion in the rodent brain

**DOI:** 10.1038/s41598-018-22268-y

**Published:** 2018-03-08

**Authors:** Amandine Jullienne, Andrew M. Fukuda, Aleksandra Ichkova, Nina Nishiyama, Justine Aussudre, André Obenaus, Jérôme Badaut

**Affiliations:** 1 Basic Sciences Department, Loma Linda University, Loma Linda, CA 92354 USA; 2 Department of Physiology, Loma Linda University, Loma Linda, CA 92354 USA; 3 CNRS-UMR 5287, University of Bordeaux, 33076 Bordeaux, France; 4 Department of Pediatrics, University of California Irvine, Irvine, CA 92697 USA

## Abstract

Aquaporins (AQPs) facilitate water diffusion through the plasma membrane. Brain aquaporin-4 (AQP4) is present in astrocytes and has critical roles in normal and disease physiology. We previously showed that a 24.9% decrease in AQP4 expression after *in vivo* silencing resulted in a 45.8% decrease in tissue water mobility as interpreted from magnetic resonance imaging apparent diffusion coefficients (ADC). Similar to previous *in vitro* studies we show decreased expression of the gap junction protein connexin 43 (Cx43) *in vivo* after intracortical injection of siAQP4 in the rat. Moreover, siAQP4 induced a loss of dye-coupling between astrocytes *in vitro*, further demonstrating its effect on gap junctions. In contrast, silencing of Cx43 did not alter the level of AQP4 or water mobility (ADC) in the brain. We hypothesized that siAQP4 has off-target effects on Cx43 expression via modification of miRNA expression. The decreased expression of Cx43 in siAQP4-treated animals was associated with up-regulation of miR224, which is known to target AQP4 and Cx43 expression. This could be one potential molecular mechanism responsible for the effect of siAQP4 on Cx43 expression, and the resultant decrease in astrocyte connectivity and dramatic effects on ADC values and water mobility.

## Introduction

Water movements in the brain are critical for cellular function by regulating cell volume and homeostasis between extracellular and intracellular compartments. Water movements are also involved in cerebrospinal fluid formation, changes in osmolarity, and in the edema process. The water channels aquaporins (AQPs), and in particular AQP4, are key players in water diffusion across plasma membranes via astrocytic endfeet. AQP4, the most abundant brain AQP, has been found to be widely expressed in astrocytes in various brain structures, with regional differences in the pattern of distribution, but with a common presence in astrocytic endfeet that are in contact with cerebral vessels^1^. Despite the use of a medley of ionic channel inhibitors, to date there are no specific inhibitors for these channels^2^. However, using RNA interference experiments with small interfering RNA (siRNA) targeting AQP4 (siAQP4), it has been shown that AQP4 is involved in water movements *in vitro* in astrocyte cultures, and *in vivo* in rat brain^3–5^. Recently, astroglial water movements induced by AQP4 have been proposed to be a driving force contributing in the paravascular clearance of interstitial solutes like amyloid-β, thus participating in the so-called “glymphatic system”^6^.

**Figure 1:**
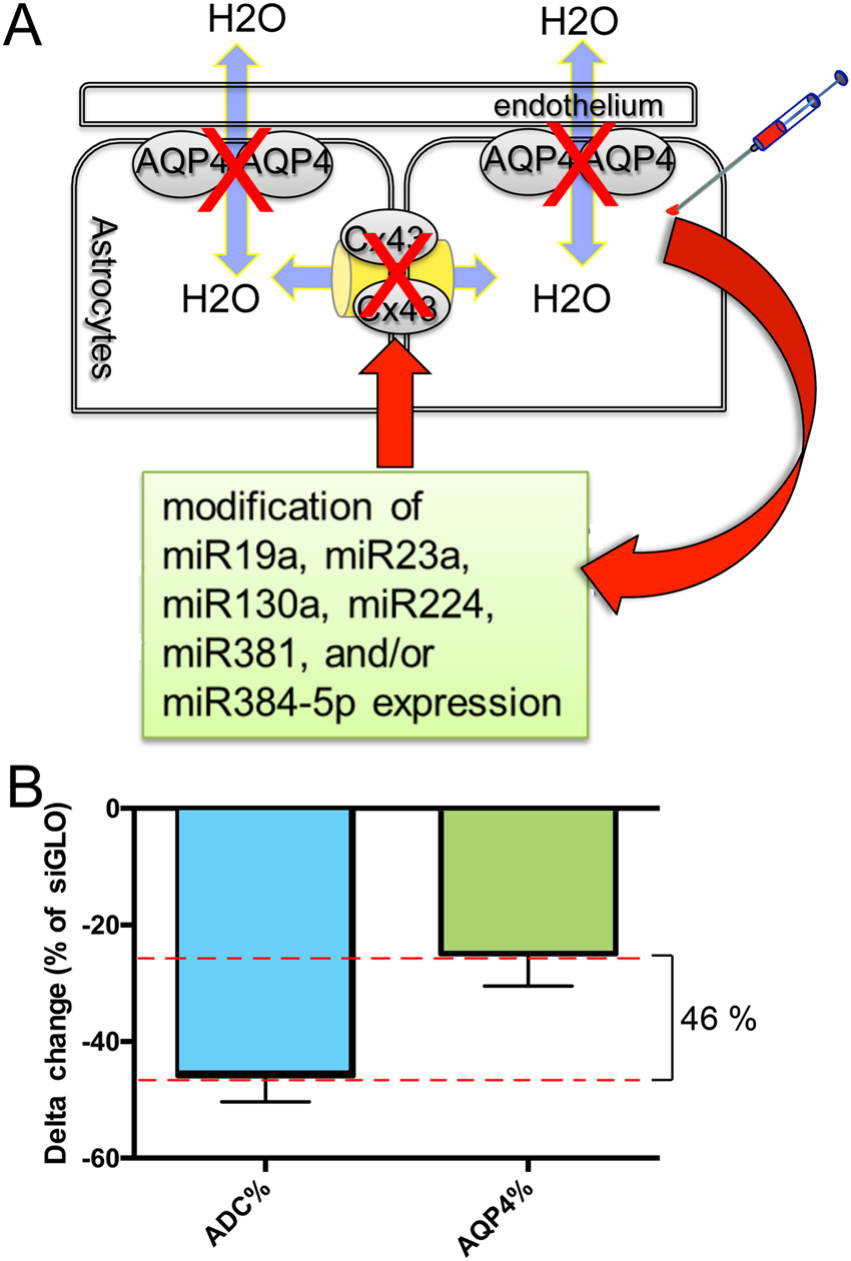
siAQP4 treatment induces a decrease in ADC values explained by decreased levels of AQP4 and Cx43 expression. (**A**) We propose that decrease of AQP4 after siAQP4 injection affects the gap junction protein Cx43 and astrocyte connectivity. Our hypothesis was that these off-target effects are mediated by miRNAs targeting both AQP4 and Cx43. (**B**) ADC values were reduced by 45.8% in the cortex 3 days after siAQP4 injection even though AQP4 levels were only decreased by 24.9%.

In our previous studies using siAQP4, we made the interesting observation that a 27% decrease in AQP4 expression elicited a 50% decrease in water mobility, as interpreted from magnetic resonance imaging (MRI) derived apparent diffusion coefficients (ADC)^3^. Such robust effects on water diffusion led us to hypothesize that siAQP4 may have other sites of action (off-target) in addition to simply decreasing the levels of AQP4 expression (see Fig. 1A). In fact, in primary astrocyte cultures it has been previously reported that siAQP4 also results in decreased expression of the gap junction protein connexin 43 (Cx43)^5^. Similarly, AQP4 knockout mice had decreased expression of Cx43^7^. Moreover, AQP4 silencing in C6 glioma cells resulted in attenuation of the increase in Cx43 expression^8^.

Cx43 and connexin 30 (Cx30) are the two primary proteins implicated in the formation of the gap junction channels in the astrocytes. These channels facilitate intercellular communication via cytoplasm-to-cytoplasm contact between astrocytes^9,10^. The term “astroglial network”^9^ is frequently used to describe those astrocytes which are interconnected with each other through gap junctions channels. Further, the absence of Cx43 and Cx30 in double knockout transgenic animals induces a loss of AQP4 channels in astrocyte endfeet, leading to a loss of blood-brain barrier integrity^11^. The molecular mechanism by which these AQP4/Cx43/Cx30 can influence each other is currently unknown.

One of the potential mechanisms for the “off-target” effects of siAQP4 could be through regulation of microRNA (miRNA). miRNAs are small (21–22 nucleotides) noncoding RNA, which have a regulatory activity in animals^12^. There are various miRNAs that target both AQP4 and Cx43 messenger RNAs (mRNAs; see Table 1) suggesting several candidates to simultaneously regulate AQP4 and Cx43 expression. We chose to study 6 miRNAs, among those most conserved between species: miR19a, miR23a, miR130a, miR224, miR381, and miR384–5p. We hypothesized that siAQP4 has off-target effects on Cx43 expression via modifications of miRNA targeting both proteins (see Fig. 1A). Supporting our hypothesis, we observed that cortical injection of siAQP4 resulted in decreased levels of Cx43 *in vivo*, along with changes in water diffusion and astrocyte connectivity. In contrast, injection of siCx43 did not alter AQP4 levels and had no effect on water movement in brain tissue. The reduction of Cx43 expression in siAQP4 treated animals relative to control animals was associated with up-regulation of miR224. The changes in miRNA expression is one molecular mechanism that could explain how siAQP4 modulates Cx43 expression in the rodent brain.

**Table 1:** Selection of miRNA targeting both AQP4 and Cx43.

	AQP4			Cx43		
	Homo sapiens	Mus musculus	Rattus norvegicus	Homo sapiens	Mus musculus	Rattus norvegicus
miR19a	●			●	●	●
	○	○	○			
miR23a	●			●	●	●
	○	○	○			
miR130a	shown to repress transcriptional activity of AQP4 M1 promoter*			●	●	●
miR224	●		●		●	●
miR381		●		●	●	●
miR384-5p	●		●		●	●
	○	○	○			

● based on *microrna.org* database.

○ based on *targetscan.org* database.

*Based on Sepramaniam *et al*.^40^.

## Results

### siAQP4 resulted in a decrease of Cx43 but not Cx30 levels

**Figure 2:**
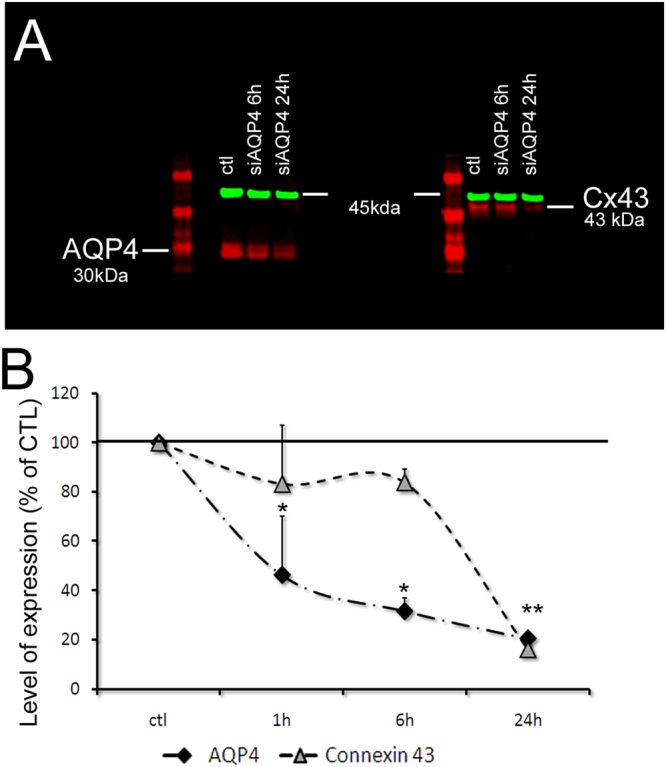
siAQP4 treatment induces decreased levels of AQP4 and Cx43 expression in primary astrocyte cultures. (**A**) siAQP4 induced a reduction of AQP4 at 6 and 24 h in primary astrocyte cultures measured by Western blot. The red band at 30 kDa corresponds to AQP4 protein (left panel) and the red band at 43 kDa is the Cx43 protein (right panel). The level of expression of both proteins is normalized to actin (in green at 45 kDa). (**B**) AQP4 shows a progressive decrease up to 24 h. Cx43 levels were decreased at 24 h, suggesting an indirect effect of siAQP4 on Cx43 expression. (*p < 0.05 compare to CTL for AQP4; **p < 0.05 compare to CTL for Cx43; values are represented as mean ± SEM).

Cortical injection of siAQP4 resulted in a 45.8 ± 4.4% decrease in ADC within the cortex as we have previously described^3^. However, decrease in protein AQP4 expression was only 24.9%± 5.5%, which does not fully explain the reduction in ADC (Fig. 1B). In primary astrocyte cultures, reduction in AQP4 expression was observed in 1 h, followed by reduction in Cx43 expression. Both proteins decreased by 80% in 24 hours (Fig. 2A). The temporal pattern of decrease was dissimilar between both proteins with a more rapid decrease in AQP4 expression compared to Cx43 (Fig. 2B).

**Figure 3:**
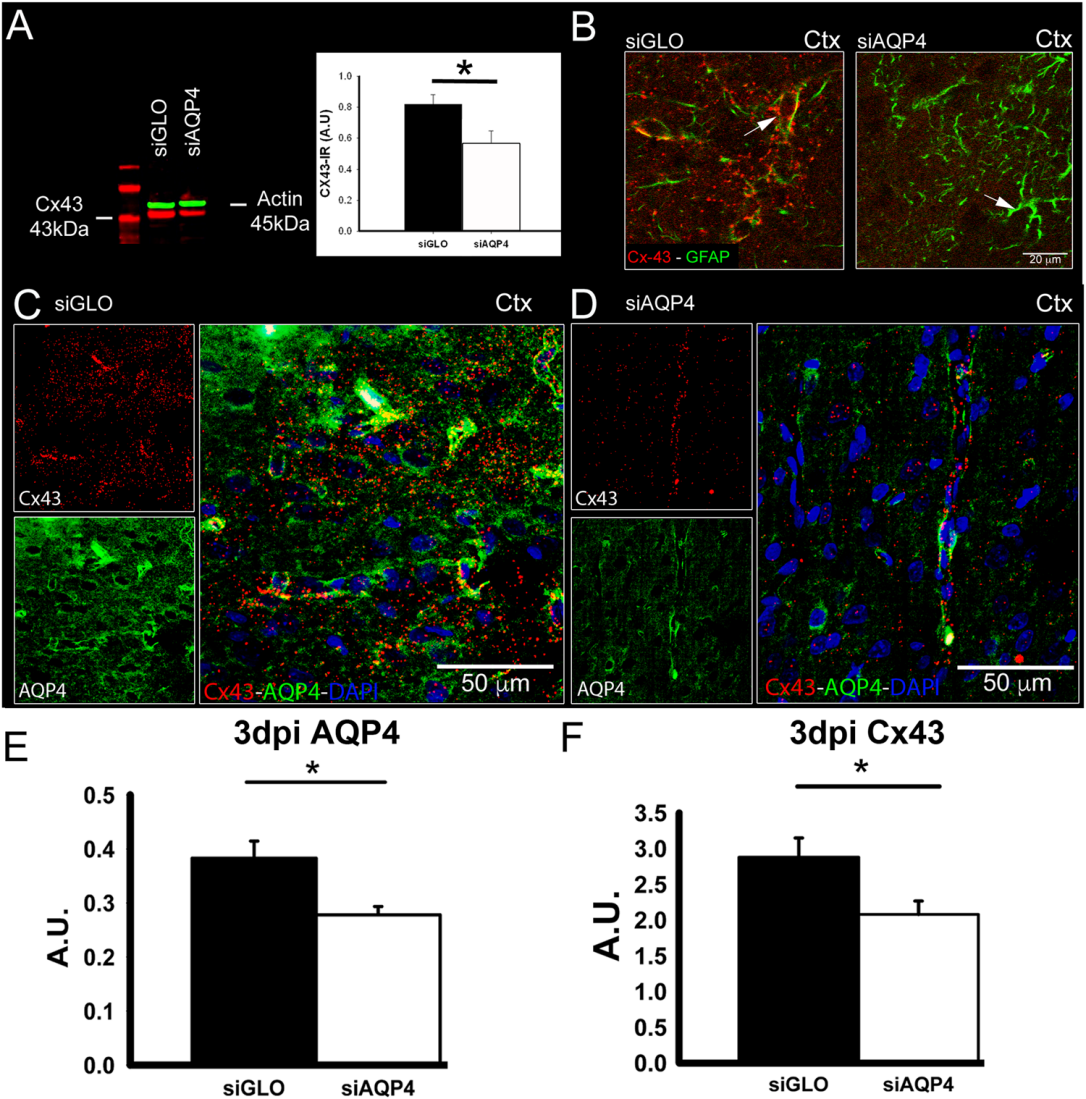
*In vivo* treatment of siAQP4 induces decreased expression of Cx43 in the cortex. Expression of Cx43 was decreased in the cortex 3 days after siAQP4 treatment as shown by Western blot assay with actin (45 kDa) used as a loading control (**A**), and by immunohistochemistry (**B**). In siGLO control animals, Cx43 protein was colocalized with GFAP in the astrocytic endfeet but was almost absent from the cortex of the siAQP4-treated rats (**B**). AQP4 and Cx43 also colocalize around blood vessels in the cortex of siGLO animals (**C**) but were both decreased after siAQP4 treatment (**D**), as shown by immunoreactivity quantification (A.U. Arbitrary Unit; **E–F**). Scale bars: B: 20 µm, D: 50 µm. *p < 0.05; values are represented as mean ± SEM.

Then, we measured the level of AQP4 expression as well as Cx43 protein changes *in vivo* from siGLO or siAQP4 experiments. Western blot experiments revealed a significant decrease of Cx43 protein levels in the ipsilateral cortex 3 days after injection (Fig. 3A). In siGLO control animals, characteristic punctuate staining of Cx43 in the cortex was observed, wrapping around the blood vessels (arrow, Fig. 3B) and co-localized with AQP4 (Fig. 3C). Cortical AQP4 expression was decreased as we previously observed^3^ (Fig. 3D,E). Animals treated with siAQP4 showed decreased AQP4 and Cx43 immunoreactivity, confirming the Western blot and demonstrating that Cx43 levels were significantly decreased (~25%) following siAQP4 treatment compared to siGLO controls (p < 0.05, Fig. 3C–F). These data are in accordance with our observations *in vitro* on astrocyte cultures, where application of siAQP4 induced a decrease in AQP4 and Cx43 expression (Fig. 2A).

**Figure 4:**
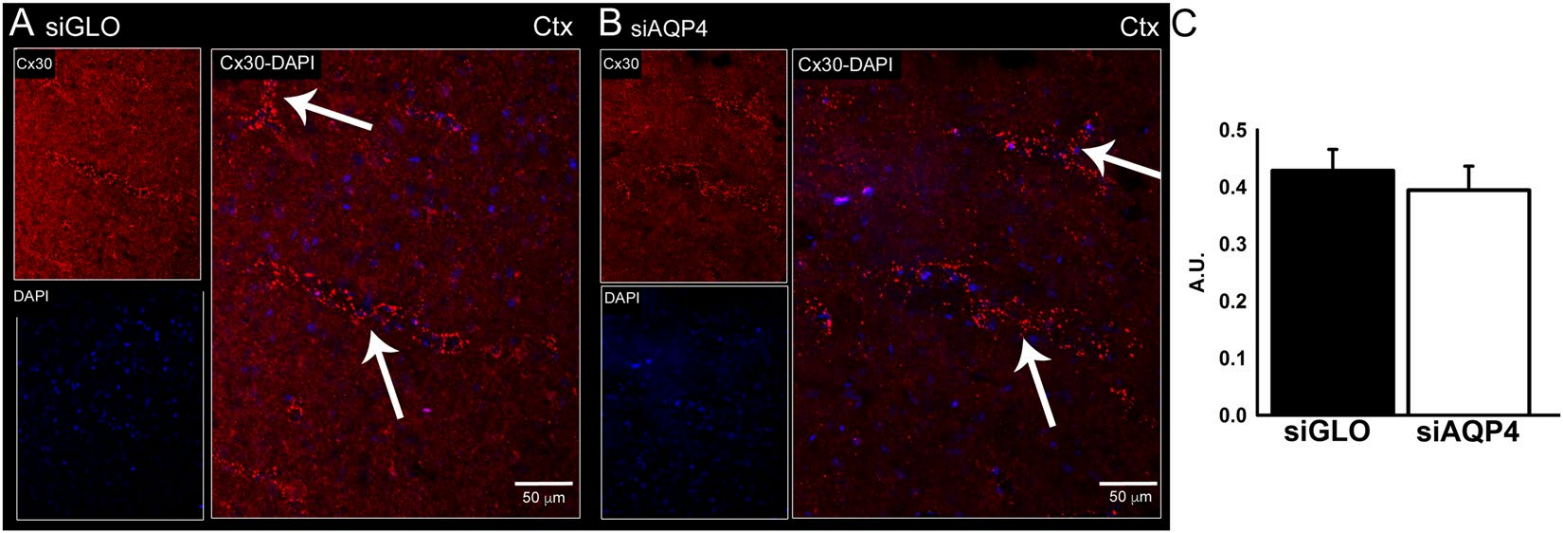
*In vivo* treatment of siAQP4 does not affect the gap junction protein Cx30. There was no difference in Cx30 staining between siGLO and siAQP4-treated animals as shown by immunohistochemistry in the cortex 3 days after injection (**A**,**B**). Quantification of Cx30 immunoreactivity in the cortex confirmed the absence of effect of siAQP4 on Cx30 expression (A.U. Arbitrary Unit). Scale bars: A, B: 50 µm.

We also evaluated if siAQP4 resulted in an overall decrease of another astrocytic gap junction, Cx30. Interestingly, the immunohistochemical localization of Cx30 revealed no difference between siGLO and siAQP4-treated animals (Fig. 4A–C). AQP4 expression selectively affected Cx43 subtype of gap junction proteins.

**Figure 5:**
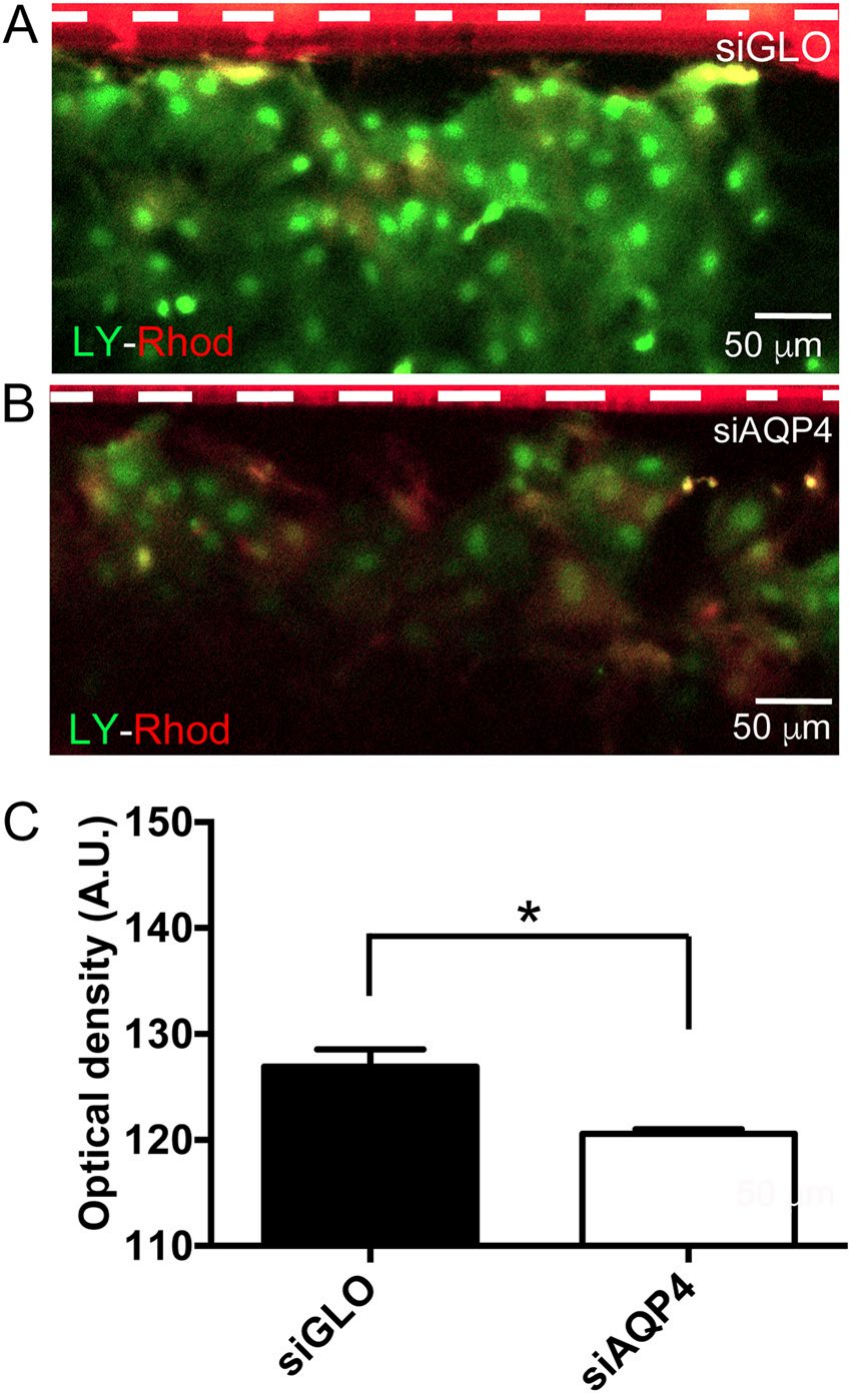
Effects of siAQP4 on astrocyte dye-coupling. (**A**) Scrape loading experiments (white dotted line) on astrocyte cultures revealed the effect of siAQP4 on astrocyte connectivity, evidenced by a decreased spread of Lucifer yellow (green) in the siAQP4-transfected astrocytes. Rhodamine B-Dextran loading (red) was similar and did not diffuse at distance from the site of scrape loading in both conditions. (**B**) Quantification of the fluorescence of Lucifer yellow confirmed the functional consequence of siAQP4-induced decrease of Cx43 (A.U. Arbitrary Unit). Scale bars: A, B: 50  µm; *p < 0.05; values are represented as mean ± SEM.

We then tested *in vitro* the functional consequences of decreased Cx43 on astrocyte connectivity after siAQP4 application using the scrape loading/dye transfer assay^13^. The decrease in Cx43 protein levels after siAQP4 transfection was accompanied by decreased astrocyte connectivity (Fig. 5). We observed prominent spreading of Lucifer yellow in the siGLO transfected cultures (Fig. 5A) indicative of an actively connected syncytium. However, in siAQP4 transfected cultures, the spread of Lucifer yellow was significantly decreased (Fig. 5B). The mean Lucifer Yellow fluorescence diffusion measured at distance from the scrape site (with a ROI placed at 10–15 μm from the red line) showed a significant decrease in siAQP4-treated (120.6 ± 0.4 A.U.) compared to siGLO control cultures (126.9 ± 1.6 A.U; Fig. 5C). In both conditions, Rhodamine B-Dextran was simultaneously loaded but did not spread, indicating that Lucifer Yellow spread through narrow gap junctions (Fig. 5A,B, Supplementary Fig. 5).

### siCx43 did not result in a decrease of AQP4 levels

**Figure 6:**
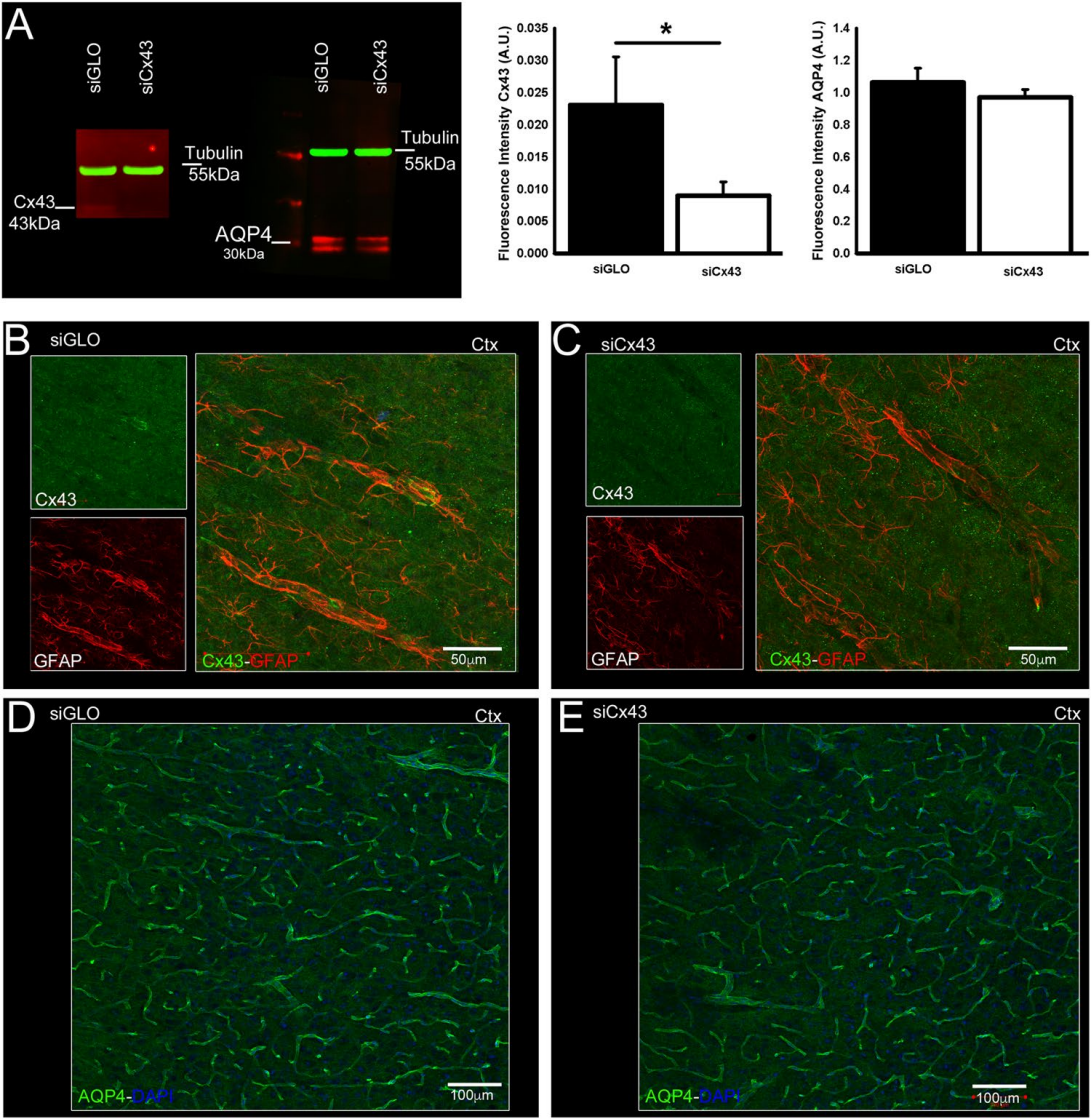
Effects of siCx43 on AQP4 and Cx43 expression. Intracortical injection of siCx43 induced a decreased expression of Cx43 and no change in AQP4 expression, as shown by Western blot with tubulin (55 kDa) as a loading control (A.U. Arbitrary Unit; **A**), and by immunohistochemistry in the cortex (**B,C** for Cx43, **D,E** for AQP4). Scale bars: B, C: 50 µm; D, E: 100 µm; *p < 0.05; values are represented as mean ± SEM.

**Figure 7:**
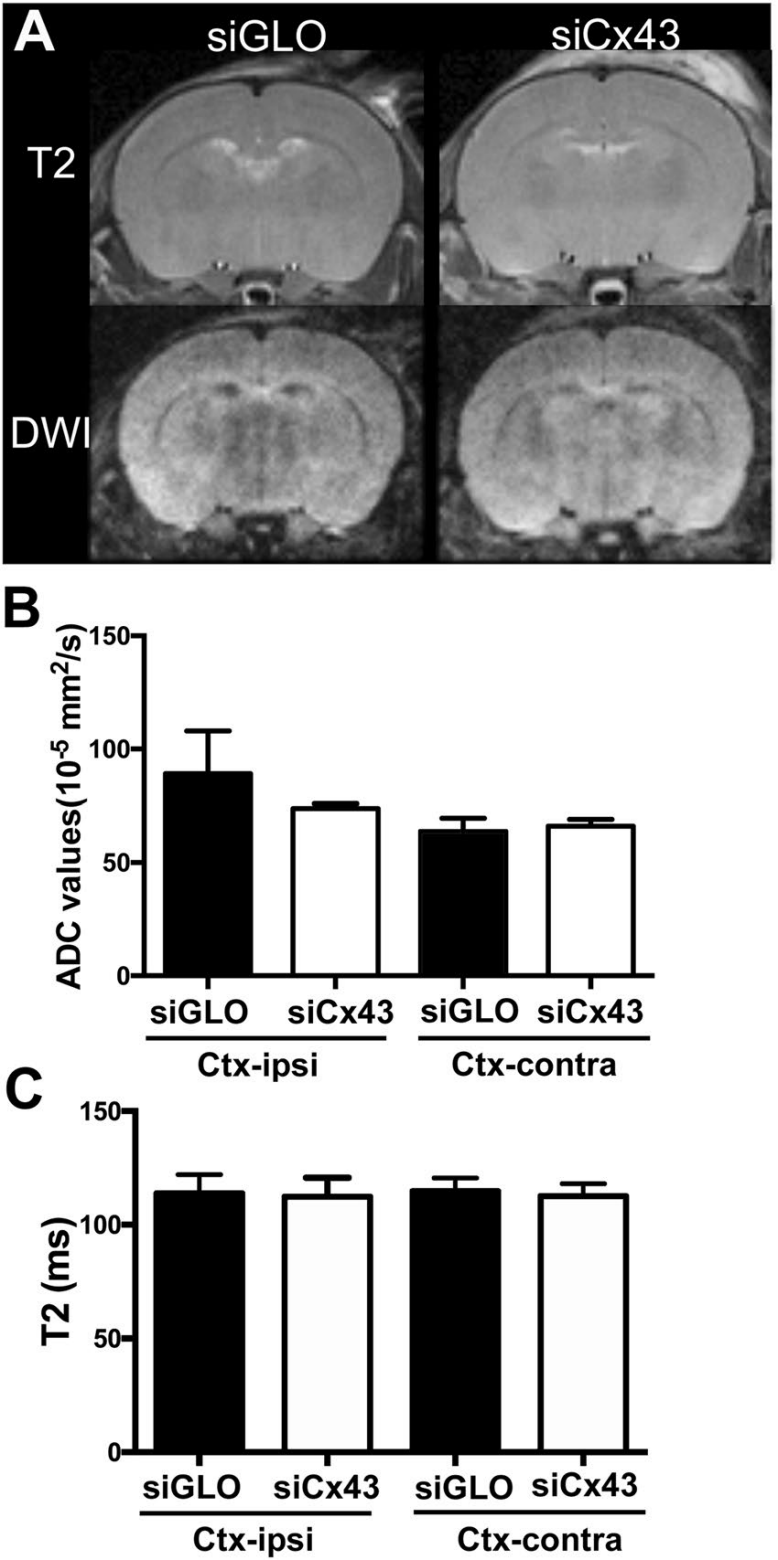
Effects of siCx43 treatment on T2 and ADC values. Representative T2WI and DWI pictures from siGLO and siCx43 treated mice (**A**). siCx43 treatment did not change the ADC values (**B**) or the T2 values (**C**) in contra or ipsilateral cortex when compared to siGLO.

To examine whether the down-regulation of Cx43 results in reciprocal down-regulation of AQP4 expression we also examined the effects of siCx43 on AQP4 expression. Immunohistochemistry and Western blot analysis for Cx43 revealed that intracortical injection of siCx43 induced a ~30% siCx43 decreased Cx43 expression by 30% (Fig. 6A–C) while AQP4 expression was not affected (Fig. 6A,D,E; Supplementary Fig. 6). To correlate the changes in AQP4 and Cx43 to brain water diffusion and water content, MRI analyses were carried out in siCx43 compared to siGLO-treated animals. In the cortex, ADC values were not significantly different between siCx43 and siGlO treated rats (p = 0.54; Fig. 7A). Similarly, T2 values were 114.39 ± 5.78 ms and 114.82 ± 9.28 ms respectively in siCx43 and siGLO groups (p = 0.96, Fig. 7B). We found no observable effects on MRI derived T2 or ADC of siCx43 treatment (Fig. 7). While siCx43 did not affect AQP4 expression as described above, a significant 31% increase in Cx30 was observed (Supplementary Fig. 7).

### siAQP4-induced down-regulation of Cx43 could be in part due to miR224 increased expression

**Figure 8:**
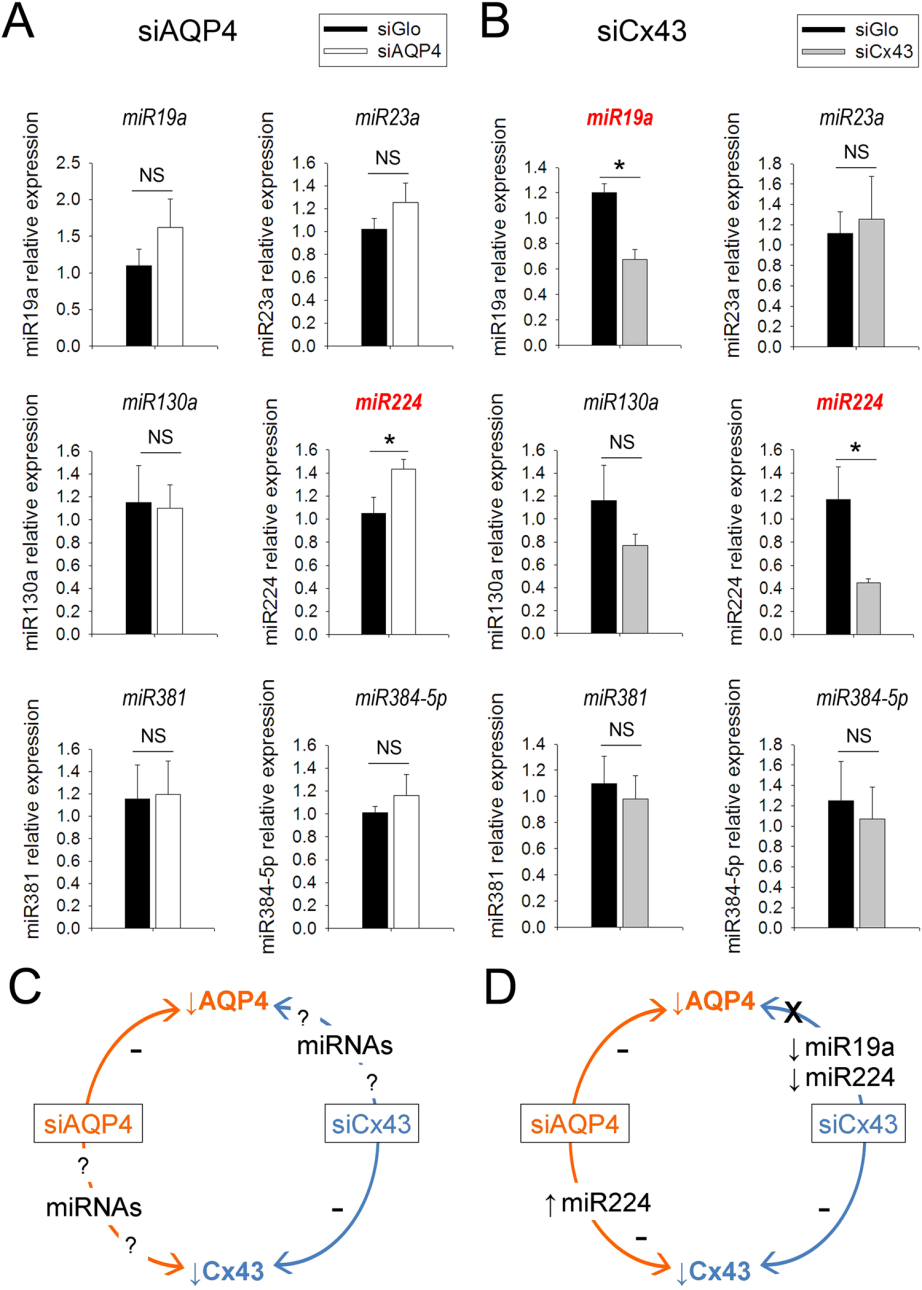
Modulation of miR19a, miR23a, miR130a, miR224, miR381 and miR384-5p expression after siAQP4 and siCx43 treatment. Relative expression of miRNA in the cortex of siAQP4- (**A**) and siCx43- (**B**) treated rat pups. RNU6-2 was used as a normalization control. Schematic representation of our hypothesis (**C**) and results (**D**) which suggest a siAQP4-induced up-regulation of miR224 which mediates the down-regulation of his target Cx43. Interestingly, siCx43 treatment induced a down-regulation of miR19a and miR224, which in turn, were not able to decrease AQP4 expression; *p < 0.05; values are represented as mean ± SEM.

To verify our hypothesis (Fig. 1A) and determine if siAQP4 had any effect on miRNAs (see Table 1), we examined the expression of the selected miRNAs in the cortex where siGLO or siAQP4 were injected using quantitative real-time PCR (qPCR). No significant difference was observed between control and siAQP4 animals for miR130a or miR381. However, we observed a significant 36% increase in the expression of miR224 in the ipsilateral cortex of siAQP4-treated animals relative to siGLO controls (Fig. 8A). Interestingly, even though not significant, miR19a, miR23a and miR384-5p exhibited an increased expression in the siAQP4-treated animals of 47%, 36% and 15%, respectively. Overall we observed that the siAQP4-induced increase of miRNAs targeting both AQP4 and Cx43 results in decreased protein expression.

We also used qPCR to examine the expression of the selected miRNAs when animals were treated with siCx43 (Fig. 8B). We found significant decreased expression of miR19a (44%) and miR224 (61%) in the ipsilateral cortex of siCx43 animals compared to siGLO control animals (Fig. 8B). Interestingly, even though not significant, miR130a, miR381 and miR384-5p also exhibited decreased expression in the siCx43-treated animals of 33%, 11% and 14%, respectively. In contrast to siAQP4 conditions, the siCx43-induced decrease of the miRNAs targeting both AQP4 and Cx43 has for consequence no change of the expression level of the protein of interest.

## Discussion

Aquaporin 4 (AQP4) is the most abundant water channel in the brain and its location on astrocytic endfeet that are in contact with blood vessels and synapses demonstrate that it is a key player in water diffusion, homeostasis and edema formation in numerous brain disorders^2^. The gap junction channels formed by connexin proteins (Cx43 and Cx30) play a central role in ionic and small signaling molecules (less than 1 kDa) by facilitating exchange between cells. The movement of the solute through the gap junctions is followed by the water movement. Further, in astrocytes, Cx43 is the most abundant connexin^14^.

AQP4 and Cx43 have been shown to share the same location on astrocytic endfeet, around blood vessels^11^. Our group showed that silencing of AQP4 by siRNA in the cortex of rat pups induces a decrease in water diffusion, as observed with reduced MRI-derived ADC values^3^. Interestingly, the decreased water diffusion appears to play a more important role than the simple down-regulation of AQP4 expression (45.8% versus 24.9%). An additional player could be Cx43 since previous studies have reported siAQP4-induced reduction in Cx43 levels in astrocyte cultures^5^ and in rat glioblastoma^8^. The regulation of Cx43 by AQP4 levels has also been evidenced *in vivo* in AQP4 knockout mice which have a decreased expression of Cx43 in ependymal cells^15^. A functional inter-relationship between AQP4 and Cx43 in astrocytes was first hypothesized by Nicchia and collaborators in an *in vitro* model^5^. Concomitant with Cx43 down-regulation, they showed that siAQP4 induced changes in astrocyte morphology through rearrangement of F-actin cytoskeleton^5^. We here confirm and extend this functional relationship between the two proteins by showing that in astrocyte cultures the siAQP4-induced down-regulation of Cx43 leads to a significant decrease in astrocytic connectivity (Fig. 5). With no overt siAQP4 effects on Cx30 expression *in vivo*, our results are consistent with previous studies showing that the coupling between astrocytes is decreased by half in Cx43-deficient mice, and abolished when Cx43 and Cx30 are absent^16^.

A new study from Katoozi and colleagues show conflicting results with an increase in connexin-labeled gap junctions in AQP4 knockout mice and a developmental effect cannot be ruled out when using total AQP4 knockout mice^17^. The authors did not observe any change in Cx30 and Cx43 mRNAs, their results suggest that this is due to an aggregation of Cx43 into gap junctions^17^.

The specific effect of siAQP4 on Cx43 but not on Cx30 suggests that there is a unique relationship between Cx43 and AQP4. Boulay and colleagues confirmed this relationship by showing that AQP4 and Cx43 expression are not modified in the brains of Cx30 knockout compared to wild type mice^18^. Cx43 and Cx30 are the two main connexins forming gap junctions in astrocytes but differences exist between the two. Cx30 has a delayed developmental onset compared to Cx43 and has lower expression^14^. Koulakoff and colleagues also found that Cx30 is not expressed in pure cortical astrocyte cultures and that the presence of neurons is required for Cx30 expression^19^. Lower expression levels of Cx30 compared to Cx43 could explain, in part, why siAQP4 has no effect on Cx30 expression.

In our experiments, we showed that the down-regulation of AQP4 induces a decrease of water movement in the cortex as seen with decreased ADC values (Fig. 1B). In contrast, the water diffusion remains unchanged after down-regulation of Cx43 by siCx43 injection (Figs 6 and 7). These results would confirm that the AQP4 and not Cx43 is critical in initiating changes in water diffusion and very likely reports water diffusion between the extracellular and intracellular compartments. A study from Hansen and collaborators supports our findings by showing that unlike AQP4-expressing *Xenopus laevis* oocytes, Cx43-expressing oocytes are not permeable to water^20^. However, the decreased level of Cx43 after siAQP4 application could contribute to the major decrease of the ADC by limiting water diffusion in the astrocyte syncytium by decreasing gap junctions and the connectivity between the cells. The decrease in ADC values in siAQP4-treated mice could be due to decreased tissue level perfusion on solute motion as reported in DWI with the use of “b” value between 0 and 200 for DWI acquisition^21^. Due to the presence of the AQP4 and Cx43 in astrocyte endfeet, the changes in the level of expression of these proteins could affect the microperfusion and values of ADC (for review^21^). However, in a variety of tissues, predominately tumors, the effects of perfusion on DWI have been estimated to be <7%^22,23^. To the best of our knowledge, there was no description of major changes for brain blood perfusion after decrease of AQP4 and Cx43. Thus, while our DWI results likely have intravoxel incoherent motion within each voxel, such effects we believe are minimal, compared to the 45.8% decrease of ADC values in siAQP4 condition relative to siGLO.

Several underlying mechanisms could be considered to explain the decreased expression of Cx43 following the siAQP4 treatment. Off-target effects of siRNA have previously been described. For example, Jackson and collaborators designed several siRNAs targeting he same gene and transfected them in HeLa cells^24^. They noticed that the transcript profiles were not target gene-specific but siRNA-specific, they even highlighted a direct silencing of non-target genes. However, off-target effects do not seem to be the case here since the Cx43 decreased expression occurs with a temporal delay compared to the AQP4 expression (see Fig. 2). Therefore, we could hypothesize that the siAQP4 has a direct effect on miRNA expression, leading to the reduced Cx43 levels. John and collaborators studied the possibility of a miRNA pathway disruption induced by *in vivo* gene silencing^25^. By using *in vivo* administration of siRNA targeting 2 different hepatocyte-expressed genes in the mouse and hamster liver, they did not observe any effect on endogenous miRNAs that are known to be expressed in hepatocytes (miR122, miR16, let-7a) and concluded that gene silencing using siRNA can be achieved without alteration of cellular miRNA biogenesis and function^25^. Our results show a change in miRNA expression after treatment with siRNA (siAPQ4 and siCx43). However, we cannot affirm that it is directly related to the siRNA injection, or if it is due to the change in AQP4 or Cx43 expression. In support to our hypothesis, a decrease in Cx43 has also been reported in AQP4 knockout animals^15^ and in neuromyelitis optica, a pathology where AQP4 expression is decreased by an anti-AQP4 antibody^26^, suggesting a direct effect of the decreased expression of AQP4 on Cx43. Evaluating miRNA expression in AQP4 knockout/Cx43 knockout mice compared to wild type mice could resolve this question. However, siAQP4 and siCx43 injections appear to affect levels of miR224 and miR19a in opposite directions with an increase in the siAQP4-treated cortex versus a decrease in the siCx43 brain. These changes correspond to the altered level of expression of AQP4 and Cx43 (Figs 5 and 8). Therefore, the level of expression of AQP4 and Cx43 possibly affects the miR224 and miR19a with an opposite effect: a decrease of AQP4 is associated with an increase of both miRNAs and decrease of Cx43 is linked with a decrease of these miRNAs (Fig. 8).

The decreased AQP4 levels concomitant with decrease in Cx43 and astrocyte connectivity in the brain likely have functional consequences on pathophysiological mechanisms: we previously showed that the use of siAQP4 affects cortical and striatal water mobility in normal physiological conditions^3^ and modifies the edema process after juvenile traumatic brain injury (TBI) in rats^27^. Astrocytes are known to be involved in spreading depression (SD): as described by Seidel and colleagues, astrocytes uptake a large amount of potassium during SD and this results in astrocyte swelling and calcium waves spreading through astrocytic networks^28^. The decrease in AQP4 levels could impact SD, in fact, Yao and collaborators showed that AQP4 knockout mice had reduced frequency and velocity of SD after epidural application of KCl compared to wild type mice^29^. Moreover, since Cx43 is an important component of the gap junctions in astrocytes and is involved in potassium and glutamate buffering, the siAQP4-induced Cx43 decrease could further alter potassium and glutamate uptake, and thus slow the propagation of calcium waves in astrocytes. The concomitant decrease of AQP4 and Cx43 would also limit the development of the second injuries after TBI as we previously observed^27^.

Furthermore, aberrant up-regulation of Cx43 and AQP4 has been reported in motor neurons in a mouse model of amyotrophic lateral sclerosis (ALS) and in ALS patients^30,31^. Similarly, astrocytic Cx43 and AQP4 have been shown to be upregulated in epilepsy, exacerbating seizures^32,33^. Therefore, interventions such as siAQP4 or miR224 and miR19a could be used as potential treatments to decrease levels of Cx43 in ALS and epilepsy.

In summary, we demonstrate for the first time that AQP4 decrease after siAQP4 induces an increase of miR224 and miR19a expression with decreases in Cx43 levels and astrocyte connectivity. These concomitant reductions may explain the mismatch between the ADC decrease and AQP4 expression. In addition, reduced Cx43 expression is not sufficient to induce decease of the ADC signal, suggesting that Cx43 in astrocytic endfeet likely do not substantially influence water mobility in the brain.

## Materials and Methods

### Animals

All animal care and experiments were conducted according to the Guidelines for Care and Use of Experimental Animals approved by Loma Linda University under the protocol # *IACUC #89024*. The manuscript was written in accordance with the ARRIVE guidelines. Sprague Dawley rat pups (Harlan, Indianapolis, IN, USA) were housed in a temperature controlled (22–25 °C) animal facility on a 12-hour light/dark cycle with standard lab chow and water *ad libitum*. Surgery was performed at postnatal day 17 (P17).

### *In vivo* siRNA Preparation and injection

*In vivo* AQP4 silencing protocol was adapted from our previous studies^3,27^. Briefly, SMART pool® containing four siRNA duplexes against AQP4 (400 ng, siAQP4, Dharmacon Research, Lafayette, CO, USA), Cx43 (400 ng, siCx43, Dharmacon Research) or non-targeted siRNA (siGLO RISC-free control-siRNA, Dharmacon Research) were mixed with Interferin® (Polyplus-transfection, Illkirch, France) diluted in a saline solution (0.9%) containing 5% glucose for a final volume of 5 μL and incubated on ice for 20 minutes for complex formation before injection.

P17 rats were anesthetized with isoflurane and placed in a stereotaxic apparatus (David Kopf Instrument, Tujunga, CA, USA). A 1 mm-diameter craniotomy over the right hemisphere, 1 mm lateral from bregma was performed prior to injection. SiAQP4 (n = 10), siCx43 (n = 10) or siGLO (n = 10) control animals were injected using a 30-gauge needle on a Hamilton syringe (1.0 mm below cortical surface). The syringe was attached to a nano-injector (Leica, Richmond, IL, USA) and a volume of 4 μL of siRNA was infused at a rate of 0.4 μL/min. After suturing, all pups received an intramuscular injection of buprenorphine (0.01 mg/kg, Tyco Healthcare Group LP, Mansfield, MA, USA) for pain relief and then placed on a warm heating pad for recovery before returning to their dams. A second injection of siRNA was repeated 2 days later for all pups using the same injection protocol.

On day 3 after MRI, one part of the animals was prepared for immunohistochemistry with PFA perfusion with n = 4 for siAQP4; n = 5 for siCx43 and n = 5 for siGLO. The second part of animals was euthanized for polymerase chain reaction (PCR) analysis. Rats were anesthetized with ketamine (90 mg/kg, IP) and xylazine (10 mg/kg, IP) and decapitated to collect brains which were then frozen on dry ice and stored at −80 °C prior to protein and RNA extraction with n = 6 for siAQP4; n = 5 for siCx43 and n = 6 for siGLO.

### Neuroimaging protocol

MRI was performed 3 days after the initial injection of the siRNA (siAQP4, siCx43 and siGLO) to observe water content and water mobility^3,27^. Rats were lightly anesthetized using isoflurane (1.0%) and imaged on a Bruker Avance 11.7 T (Bruker Biospin, Billerica, MA, USA)^27^. Two imaging data sets were acquired: 1) a 10 echo T2- and 2) a diffusion weighted imaging (DWI) sequence in which each sequence collected 20 coronal slices (1 mm thickness and interleaved by 1 mm). The 11.7 T T2 sequence had the following parameters: TR/TE = 2357.9/10.2 ms, matrix = 128 × 128, field of view (FOV) = 2 cm, and 2 averages. The DWI sequence had the following parameters: TR/TE = 1096.5/50 ms, two b-values (116.960, 1044.422 s/mm^2^), matrix = 128 × 128, FOV = 2 cm, and 2 averages.

T2 and apparent diffusion coefficient (ADC) values were quantified using previously published protocols^3^. Regions of interest (ROIs) were placed on MR images at the site of the injection on a single coronal slice (1 mm thick) using Cheshire (Parexel International Corp. Waltham, MA, USA). T2 maps were generated and ADC maps were calculated using a linear two-point fit. Four primary ROIs within ipsi and contralateral hemispheres (cortex and striatum) were delineated on T2WI with ipsi-cortex (23 pixels); contra-cortex (40 pixels); ipsi- and contra-striatum (41 pixels each). These ROIs were overlaid onto corresponding T2 and ADC maps and the mean, standard deviation (SD), number of pixels and area for each ROI were extracted. The MRI analysis was performed by two blinded readers without knowledge of the treatment. The inter-rater differences in the results obtained by the readers was 2% in siGLO-treated animals and 7% in the siAQP4-treated animals.

### Astrocyte primary cultures

Primary cortical astrocyte-enriched cultures were prepared from C57BL/6 mouse pups at P0 to P2 with adapted protocol from a previous publication^34^. Following isolation of cortices and removal of the meninges, mechanical dissociation with various sizes of needles was performed to isolate the cells; then they were suspended in DMEM supplemented with 10% fetal calf serum (FCS; Life Technologies, Thermo Fisher Scientific, Waltham, MA, USA) and antibiotic-antimycotic (Life Technologies, Thermo Fisher Scientific). Cells were seeded on 6-well plates for a growing period of 21 (±2) days at 37 °C and 5% CO_2_. Medium was changed every 2–3 days. Glial fibrillary acid protein (GFAP) staining was used to demonstrate a pure astrocyte culture^3^.

### *In vitro* siRNA transfections

*In vitro* siAQP4, and siGLO transfection was adapted from a previous publication^3,35^. Custom SMART pool containing small interference RNA duplexes against AQP4 (20 nmol/L, siAQP4, Dharmacon Research), was mixed with INTERFERin (Polyplus-transfection) diluted in NaCl with 5% glucose for a final concentration of 100 nM. For controls, non-targeted 100 nM siRNA (5 nmol/L, siGLO, RNA-induced silencing complex (RISC)-free control siRNA, Dharmacon Research) was used to monitor nonspecific effects of the siRNA. siCx43 (5 nmol/L, Dharmacon Research) was prepared using the same protocol to final concentration of 20 nM. The mixture was incubated for 5–10 minutes at room temperature for complex formation and then added to the cells. For each experiment, specific silencing was measured by Western blot at 6 h, 24 h and 4 days after transfection^3^. The experiments were repeated 3 times with 3 wells per timepoint and condition.

### Scrape loading/dye transfer assay

The gap junctional connectivity was assessed using scrape loading. The protocol was adapted from a previous publication^13^. Briefly, astrocyte cultures in 6-well plates were washed with PBS containing calcium and magnesium and subsequently incubated with a PBS solution without calcium and magnesium (the presence of calcium prevents opening of the gap junctions). Scrape-loading was performed with a razor blade in the calcium-free solution containing 0.5 mg/ml Lucifer yellow (dilithium salt, Sigma-Aldrich, Buchs, Switzerland) and 0.5 mg/ml Rhodamine B-Dextran (30 kDa, Sigma-Aldrich) as a control. The dye transfer inside the cells was allowed for 5 min and afterwards the wells were washed several times with PBS. Junctional permeability was measured 8 min after scraping by taking four successive photomicrographs per trial using the MorphoStrider software (Explora-Nova, La Rochelle, France) and an inverted epifluorescence microscope (BX-41 Olympus, Volketswil, Switzerland) equipped with appropriate filters. The surface area of the fluorescence diffusion was measure for each group with the Image J software (NIH) on 3 different regions of interest repeated in 3 wells for each experimental condition and repeated 3 times.

### Western blot analysis

Astrocyte cultures were harvested in cells lysis buffer (RIPA buffer, Buchs, Sigma-Aldrich, Switzerland) with protease inhibitor cocktail tablets (PIC, Roche, Basel, Switzerland) at 6 h, 24 h and 4 days. After imaging, four animals per experimental groups were freshly dissected at 3 days post siRNA injection. Cortical tissue adjacent to the site of injection was collected and frozen for Western blot analysis as previously published^3,36^. Cells and tissues were placed in a tube with RIPA buffer with PIC and sonicated for 30 s and stored at −20 °C. These samples were then assayed for total protein concentration by bicinchoninic acid assay (BCA, Pierce Biotechnology Inc., Rockford, IL, USA). Ten micrograms of protein were then subjected to SDS polyacrylamide gel electrophoresis on a 4–12% polyacrylamide gel (Nupage, Invitrogen, Carlsbad, CA, USA). Proteins were then transferred to a polyvinylidene fluoride membrane (PerkinElmer, Rodgau, Germany). The blot was either incubated with a polyclonal antibody against AQP4 (1:2000, Alpha Diagnostics, San Antonio, TX, USA), or a rabbit polyclonal antibody against Cx43 (1:1000, Abcam, Cambridge, MA, USA) or Cx30 (1:1000, Abcam) and a monoclonal antibody against tubulin (1:25,000, Sigma-Aldrich) in Odyssey blocking buffer (LI-COR Bioscience, Germany) for 2 h at room temperature. After washing in PBS for 3 × 10 min, the blot was incubated with two fluorescence-coupled secondary antibodies (1:10,000, anti-rabbit Alexa-Fluor-680 nm, Molecular Probes, OR, USA, and anti-mouse infra-red-Dye-800 nm, Roche, Germany) for 2 h at room temperature. After washing in PBS, the degree of fluorescence was measured using an infra-red scanner (Odyssey, LI-COR Biosciences, Lincoln, NE, USA) as previously published^27^. Quantification was performed blindly by two experimenters. The value of AQP4, Cx43 and Cx30 was normalized to tubulin and compared between siAQP4, siCx43 and siGLO-treated condition at each timepoint.

### Immunohistochemistry and Image Analysis

At 3 days, one part of the animals was transcardially perfused with 4% paraformaldehyde at 4 °C, brains were extracted and put in 30% sucrose before freezing, then stored at −20 °C. Coronal sections were cut at 20 μm thickness on a cryostat (Leica, Richmond, IL, USA) and mounted on slides for immunohistochemical analysis^37,38^. The primary antibodies used were: rabbit anti-AQP4 (1:300, Chemicon, Temecula, CA, USA) and mouse anti-AQP4 (1:100, AbD Serotec-BioRad, Hercules, CA, USA)^39^; rabbit anti-Cx43 (1:100, Invitrogen), rabbit anti-Cx30 (1:100, ThermoFisher Scientific), and chicken anti-glial fibrillary acid protein (GFAP, 1:1000, Millipore, Temecula, CA, USA). Secondary antibodies were used at 1:1000 as appropriate for each primary antibody (all secondary antibodies from Invitrogen). Negative control staining where the primary antibody or secondary antibody was omitted showed no detectable labelling.

### Selection of candidate miRNAs targeting AQP4 and Cx43 mRNAs

To determine miRNA targeting both AQP4 and Cx43 mRNA, we used literature^40^ and databases (targetscan.org and microrna.org). We chose the most conserved miRNAs between species and selected 6 miRNAs: miR19a, miR23a, miR130a, miR224, miR381, and miR384–5p (see Table 1).

### RNA extraction

Total RNA was isolated from 50–100 mg of fresh frozen ipsilateral cortex using TRIzol® reagent (Invitrogen) and chloroform. RNA was precipitated with isopropanol, washed with 75% ethanol, resuspended in RNase free water and quantified with a spectrophotometer (NanoDrop 2000, Thermo Scientific).

### Reverse transcription and real-time PCR for miRNA detection

Reverse transcription for quantitative real-time PCR was performed using the miScript II RT kit (Qiagen, Valencia, CA, USA) on a T100* Thermal Cycler (BioRad, Hercules, CA, USA). Real-time PCR were performed using the miScript SYBR® Green PCR kit (Qiagen) and the *Rn_miR19a, Rn_miR23a, Rn_miR130a, Rn_miR224, Rn_miR381* and *Rn_miR384-5p* miScript Primer Assays. Assays were run in triplicate on the *iCycler IQ5* real-time PCR detection system (BioRad). MiRNA expression levels were normalized using the miRNA RNU6-2. MiRNA expression was calculated as follows: relative miRNA expression = 2^−(Ct miRNA – Ct RNU6-2)^.

### Statistics

One way ANOVA was used for immunohistochemistry analysis to compare the mean between siGLO and siCx43 group, siGLO and siAQP4 group, siGLO and siCx30 groups. Two-way repeated measures analysis of variance with a post hoc Bonferroni test was used for MRI data. Student t-tests were used for PCR analysis. A p value less than 0.05 was considered to be statistically significant.

### Data availability

The datasets generated and analyzed during the current study are available from the corresponding author on reasonable request.

## Electronic supplementary material

Supplementary information
