# Supplementary material for: Modulating the water channel AQP4 alters miRNA expression, astrocyte connectivity and water diffusion in the rodent brain
Source: Sci Rep. 2018 Mar 8;8:4186. doi: 10.1038/s41598-018-22268-y (PMC5843607; doi:10.1038/s41598-018-22268-y)
Supplement: Supplementary file 1 — Supplementary information [file 41598_2018_22268_MOESM1_ESM.pdf]

Modulating the water channel AQP4 alters miRNA expression, astrocyte connectivity and water diffusion in the rodent brain

Amandine Jullienne[1,2#], Andrew M Fukuda[1,2#], Aleksandra Ichkova[3], Nina Nishiyama[2], Justine Aussudre[3], André Obenaus[1,4], Jérôme Badaut[1,2,3]*

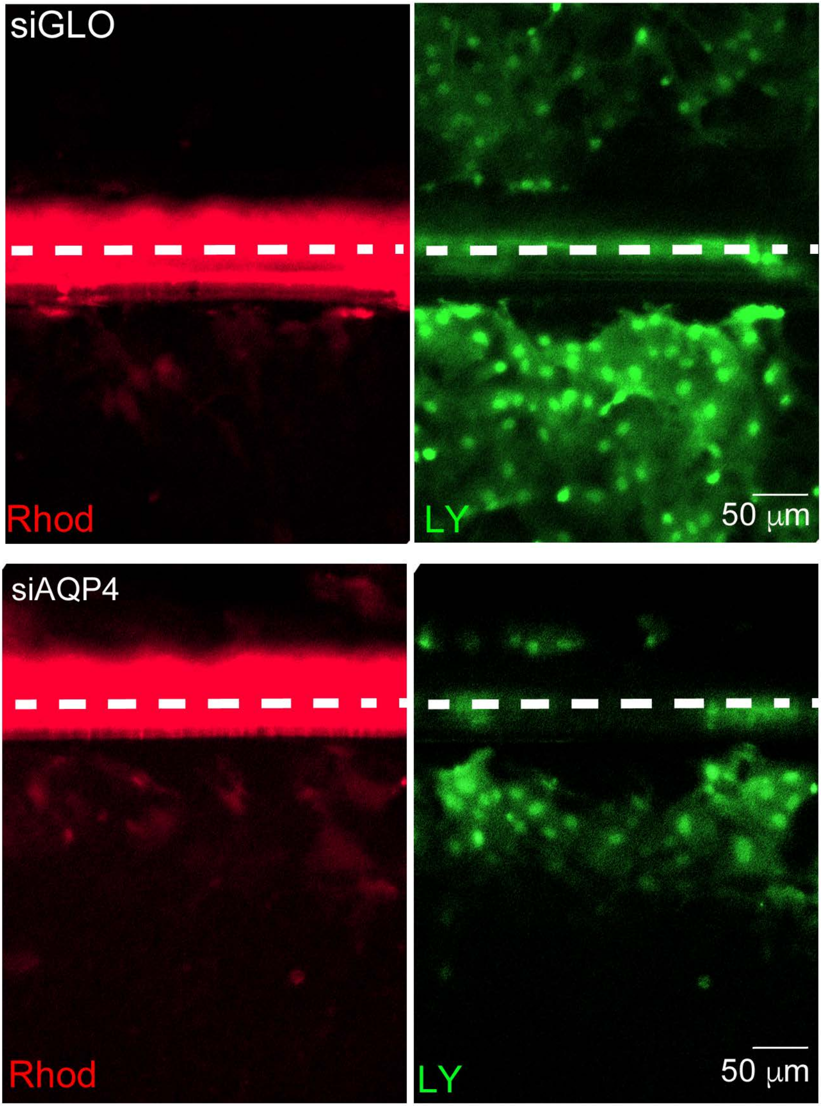
siGLO
Rhod
LY
50 μm
siAQP4
Rhod
LY
50 μm

Supplementary Figure 5: “Scrape loading experiments (white dotted line) on astrocyte cultures revealed the effect of siAQP4 on astrocyte connectivity. Lucifer yellow (LY) spreading was decreased in the siAQP4-transfected astrocytes. Rhodamine B-Dextran (rhod) loading was similar and did not diffuse at distance from the site of scrape loading in both conditions. Scale bars: 50 µm

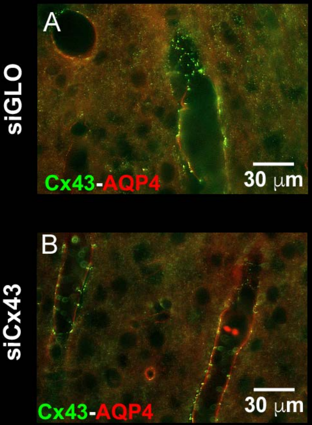
siGLO
A
Cx43-AQP4
30 μm
siCx43
B
Cx43-AQP4
30 μm

Supplementary Figure 6. Effects of siCx43 on AQP4 and Cx43 expression.

Double immunolabeling of AQP4 (red) and Cx43 (green) after intracortical injection of siGLO (A) and siCx43 (B) showed a decreased of Cx43 staining (green) and no major change in AQP4 expression (red). Scale bars: 30μm.

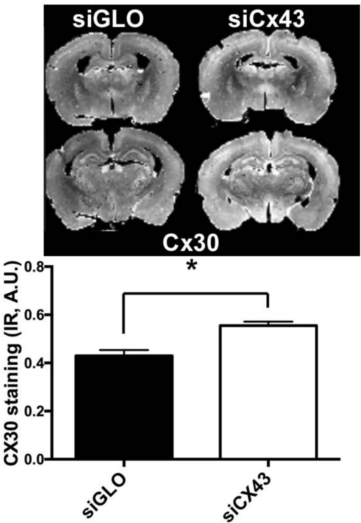

siGLO
siCx43
Cx30
*
CX30 staining (IR, A.U.)
0.8
0.6
0.4
0.2
0.0
siGLO
siCX43

Supplementary Figure 7. Effects of siCx43 on Cx30 expression.

Intracortical injection of siCx43 induced an increased expression of Cx30 as shown by infra-red immunohistochemistry (top of the figure) and on the quantification.
